# Rupture of urinary bladder: a case report and review of literature

**DOI:** 10.1186/1757-1626-2-7004

**Published:** 2009-05-14

**Authors:** Jamil Ahmed, Ismail H Mallick, Syed Muzaffar Ahmad

**Affiliations:** Department of General Surgery, Scunthorpe General HospitalScunthorpe DN15 7BHUK

## Abstract

**Introduction:**

Spontaneous rupture of the urinary bladder is a rare event. Patients usually present with features of peritonitis and diagnosis is usually made at operation. The morbidity and mortality rate is very high in these groups of patients.

**Case presentation:**

We present a case of a 47-year-old caucasian woman who was known to have transitional cell carcinoma of the urinary bladder who presented with features of peritonitis. An exploratory laparotomy revealed free perforation of the urinary bladder. The perforation was closed. However, on the second post-operative day she started draining urine from the abdominal drain and was taken back to the operating theatre. The stitches in the urinary bladder had given off and she underwent radical cystectomy along with double barrel cutaneous ureterostomies. Peritoneal biopsies revealed disseminated transitional cell carcinoma in the peritoneum. She made a slow postoperative recovery.

**Conclusion:**

Perforation of the urinary bladder should be considered in patients presenting with peritonitis particularly with a previous history of urinary bladder cancer.

## Introduction

Rupture of the urinary bladder is an uncommon and life threatening event. Prompt diagnosis followed by surgical intervention is the key for successful outcome. Often, there are obscurities in establishing exact diagnosis preoperatively which may lead to a very high mortality rate. It is also true that such patients are in advanced stage of their disease, a very few survive more than one year following diagnosis.

## Case presentation

A 47-year-old Caucasian woman presented as an emergency with generalized abdominal pain. She was diagnosed with a grade 3 transitional cell carcinoma of the postero-lateral wall of the urinary bladder, which was resected endoscopically seven months ago. This was followed by adjuvant intravesical mitomycin. She does not have any co-existent medical problems. She used to smoke 30 cigarettes per day. Her mother died due to metastatic urinary bladder cancer.

Clinical examination did not reveal any jaundice or anaemia. She was in obvious distress with abdominal pain. Her observations were as follows: temperature 37.8°Celsius, blood pressure was 110/70 mmHg, pulse 120 beats/min with a respiratory rate of 20/min. Abdominal examination revealed features suggestive of generalized peritonitis. Laboratory tests showed haemoglobin 11.4 g/dL, white cell count 34,300 cells/mm3 of blood, C-reactive protein > 300 and amylase 15U/L. The rest of her blood results were normal. Plain radiographs of chest and abdomen did not reveal any signs of perforation or intestinal obstruction. A urinary catheter was inserted which drained 1100 ml straight away and no further output. She was promptly taken for an exploratory laparotomy which revealed 500 ml of urine intraperitoneally with free perforation of the urinary bladder. The perforation was closed with interrupted Vicryl® (Ethicon, Edinburgh, UK) 3/0 stitches. A thorough washout was performed. An abdominal drain was inserted and the wound closed. On the second post-operative day the abdominal drain was draining urine and she was taken back to the operating theatre. The stitches in the urinary bladder had given off and she underwent radical cystectomy along with double barrel cutaneous ureterostomies. Peritoneal biopsies revealed disseminated TCC in the peritoneum. She made a slow postoperative recovery.

## Discussion

Spontaneous rupture of the urinary bladder is an uncommon and life threatening event. Prompt diagnosis followed by surgical intervention is the key for successful outcome. Often, there are obscurities in establishing exact diagnosis preoperatively leading to a very high mortality rate. It is also true that such patients are in advanced stage of their disease, a very few survive more than one year following diagnosis.

Studies and case reports in the literature were identified by PubMed search between the years 1967-2007 using the following free text keywords: bladder, carcinoma, spontaneous, perforation, and peritonitis from the year 1967 in the English literature. (Table 1)

**Table 1.** Shows the reports of previous case reports and series of patients with perforation of the urinary bladder with details of the site of perforation, pathology and outcome

No	Year	Author	Sex	Age	Site	Pre/Intra-op diagnosis	Histology	Outcome
1	2002	Jayathillake A [12]	F	72	? Right side of bladder wall	P	SCC*	Died in 10 days
2	2001	Basavaraj DR [8]	M	78	Not mentioned	P	TCC*	Recovered
3	2001	Goel A [13]	M	55	Dome of the bladder	I	SCC	Recovered
4	2000	Valero Puerta J [14]	M	73	Anterior wall	P	TCC	Died in the same admission
5	1998	Atalay AC [4]	F	75	Dome of bladder	I	TCC*	Died in 20 days
6	1998	O'Neill GF [15]	M	46	Vault anteriorly	I	TCC	Recovered
7	1995	Addar MH [6]	F	40	Dome of bladder	I	Firosis	Recovered
8	1994	Martínez Jabaloyas JM [16]	M	45	Not applicable	P	TCC	Died n 8 months
9	1993	Rasmusen JS [9]	F	65	Posterior wall of bladder †	I	TCC*	Died in 4 month
10	1992	Gough M [11]	F	77	Dome of bladder	I	SCC	Recovered
11	1989	Wujanto R [5]	F	79	Posterior wall (assumed)	I	SCC	Died in 24 days
12	1988	Budd JS [2]	F	79	Vault of bladder	I	TCC*	Died after some months
13	1983	Huffman JL [1] (Three cases)	M	30	Posterior bladder wall	I	Inflammation and fibrosis	Recovered
			M	73	Dome of bladder	P	Hemorrhagic necrosis and Inflammation	Recovered
			F	49	Posterior bladder wall	I	SCC*	Recovered
14	1981	Jenkinson LR [17]	F	73	Bladder	I	SCC*	Died in 21 days
15	1967	Glashan RW [3] (Two cases)	F	43	Right side of bladder	I	SCC	Recovered
			M	38	Vault of bladder	I	TCC	Recovered

**Abbreviations:** M- male, F- Female, I-Intraoperative; P- Pre-operative, TCC- transitional cell Carcinoma, SCC- squamous cell carcinoma, * - poorly differential, †-this patient had neobladder.

The most common causes of atraumatic rupture of urinary bladder are chronic inflammation, bladder outflow obstruction and cancer [1]^.^ Budd JS [12] in 1988 reported his case as first ever reported case of spontaneous rupture of the female bladder associated with a truly transitional cell carcinoma (TCC) [2], Albeit, Glashan RW in 1967 has reported his case as first ever perforation in the male population in a patient with TCC [3]. Glashan RW attributed this to anatomical feature of the male urethra making the male bladder more liable to distention with consequent perforation.

Clinically most of patients presented with lower abdominal pain with associated symptoms of dysuria, unable to void, anuria and haematuria. In the majority of cases the symptoms of urinary tract infection (UTI) were the initial complaints and this was later accompanied by peritonism. A clinical diagnosis of acute abdomen was made in all the cases, which were reviewed.

An accurate preoperative diagnosis of urinary bladder rupture was made only in two out of fifteen case reports [4,5]. Interestingly, many of these cases were initially treated conservatively with catheterisation and antibiotics.

The site of the perforation was dependant on where the tumour was situated as shown from previous studies. The possible pathogenesis of bladder rupture in bladder cancer is precipitation of perforation on the weakened body wall by the tumour. Although the most frequent location for intraperitoneal perforation was the dome or the posterior wall of the bladder [6].Computed Tomography (CT) scan of the abdomen and urinary cystogram can yield diagnostic results [7]. Cystogram is the diagnostic test of choice, though false negative cystogarphy with bladder perforation is not uncommon. Lowe et al reported the successful use of CT scan in confirming the diagnosis when cystography is negative or equivocal [7]. Combination of CT and cystography is an accurate non-invasive method for assessing bladder pathology especially in the patients having suspension of bladder perforation.

Basavaraj et al recommend that conservative management of spontaneous perforation of the bladder following radiotherapy, with antibiotic and prolonged catheterization is a better option than surgical intervention [8].

Transitional cell carcinomas (TCC) account for about 90% of all bladder tumours, where as squamous cell carcinoma comprise less than 5%. In this review we found eleven cases of TCC and eight cases of SCC which have perforated suggesting squamous cell carcinomas are more likely to perforate than TCC. These findings were also concurred by Rasmusen JS [9].

Chronic cystitis in the presence of indwelling catheters or calculi is associated with increases risk of SCC (non-bilharzail type) of the urinary bladder [10].

The prognosis of spontaneous bladders rupture due to carcinoma is very poor. Most of patients, in this review, died within months ranging from 10 days to 8 months. The mortality rate can range from 25% to 80% depending up time of diagnosis [6,11].

## Conclusion

Patients with rupture of urinary bladder usually present with symptoms and signs of peritonitis. A history of unexplained urinary tract symptoms prior to the acute episode is not uncommon in most of these patients. A high index of suspicion is essential in the presence of urinary symptoms and signs suggestive of peritonitis in a patient with bladder cancer. Rupture of urinary bladder must be included in the differential diagnosis of acute abdomen. This is a rare but potentially fatal condition with a mortality rate more then 80%.

## List of abbreviations

CT: Computerised tomography
SCC: Squamous cell carcinoma
TCC: Transitional cell carcinoma
UTI: urinary tract infection
